# Assessing the financial resources required to establish convalescent plasma collection programmes across Europe in response to a pandemic

**DOI:** 10.1111/trf.70323

**Published:** 2026-10-01

**Authors:** Elizabeth A. Stokes, Abigail A. Lamikanra, David J. Roberts, Lise J. Estcourt, Pierre Tiberghien, Jose Leal

**Affiliations:** ^1^ Economics of Population Health (EPH) Research Centre, Nuffield Department of Population Health University of Oxford Oxford UK; ^2^ NHS Blood and Transplant Oxford UK; ^3^ Radcliffe Department of Medicine University of Oxford Oxford UK; ^4^ Etablissement Francais du Sang La Plaine‐St Denis France; ^5^ Université de Franche‐Comté EFS, INSERM, UMR Right Besançon France; ^6^ Nuffield Department of Primary Care Health Sciences University of Oxford Oxford UK

**Keywords:** blood establishment, convalescent plasma, costs, COVID‐19, Europe

## Abstract

**Background:**

During the COVID‐19 pandemic, convalescent plasma (CP) was investigated as a potential treatment. We assess the financial resources required to implement a COVID‐19 CP (CCP) programme and provide recommendations on how best to prepare for collection and use of CP in future pandemics.

**Study Design and Methods:**

We mapped pathways for collecting and manufacturing CCP using a UK blood establishment as a test case, and identified the resources required at each stage to provide high‐titre (HT) CCP units. A top‐down micro‐costing approach and an economic model were used to establish the cost per HT CCP unit. Data collection questionnaires were later prepared to acquire data from 21 countries across Europe, facilitate comparisons and estimate costs per HT CCP unit using a top‐down gross‐costing approach. Costs are reported in Euros adjusted for cost‐of‐living differences.

**Results:**

The key cost driver for England was the cost of CCP collection. Nine countries responded, four with existing infrastructure for apheresis plasma collection at the start of the pandemic and five without. Four countries provided sufficient data to estimate the cost per HT CCP unit, which varied between €198–€265 for countries with pre‐existing apheresis plasma collection and €807–€1749 for those without.

**Discussion:**

Countries had marked differences in the scale of CCP collection, and methods of selecting and measuring HT CCP, which contributed to the variation in expenditure observed. Costs were significantly lower in countries with existing infrastructure for apheresis plasma collection and where donors were tested for anti‐SARS‐CoV‐2 antibody titre before attending plasma collection.

**Abbreviations**
AfC: Agenda for Change
CCP: COVID‐19 convalescent plasma
CP: convalescent plasma
HT: high‐titre
nAb: neutralizing antibody
NHSBT: NHS Blood and Transplant
PCR: polymerase chain reaction
PPPs: purchasing power parities
RCTs: randomized controlled trials
SCB: SoHO Coordination Board
SoHO: standards of quality and safety for substances of human origin
SUPPORT‐E: supporting high quality evaluation of COVID‐19 convalescent Plasma throughout Europe

## 1 INTRODUCTION

COVID‐19, caused by viral infection with SARS‐CoV‐2, emerged in late 2019 and spread very quickly across the globe. In the next 2 years, more than 58 million cases and 900,000 deaths were reported in the EU/EEA alone.^1^ When COVID‐19 became a global pandemic in 2020, one of the potential treatments investigated was convalescent plasma (CP). This is plasma collected from recovered COVID‐19 patients, containing antibodies against SARS‐CoV‐2 antigens that could neutralize the virus in vitro and potentially in vivo. Platform randomized controlled trials (RCTs) were established in the UK^2^ and globally with various international study sites.^3^ These trials compared the outcomes of treatment using COVID‐19 convalescent plasma (CCP) with standard treatment of patients hospitalized with severe/moderate COVID‐19. If CCP was found to be an effective treatment, then suddenly there would be high demand for this product, and countries would need to scale up production. Therefore, before trial results were known, many countries set up CCP collection programmes, which required significant resources. While previous studies have reported on the development and implementation of CCP programmes, for example in the USA,^4^, ^5^ Japan^6^ and Brazil,^7^ and Russell et al. developed a demand and supply model for CCP in the US,^8^ we are not aware of any studies assessing the costs of implementing a CCP programme across several countries.

While there was uncertainty at the time of how CCP would be used therapeutically, there was strong interest in identifying the resources required to set up and run CCP programmes across Europe. This included the costs associated with collecting and testing for CCP with high concentrations of neutralizing antibodies—high titre (HT) CCP, since HT CCP was anticipated to have greater efficacy against COVID‐19.

Our aim was to map the pathways to provide HT CCP units and cost them across Europe. We identified key cost drivers, which could then inform the use of CP to treat new and emerging infections. Finally, we compared the total volume of CCP units collected, existing infrastructures for plasma collection at the start of the pandemic, and equipment prices to better understand potential variation in costs. Cost evidence is critical for future pandemic preparedness, since resource allocation decisions depend on such models.

## 2 METHODS

We used the largest provider of blood products in the UK, NHS Blood and Transplant (NHSBT) in England, as a test case to first map the pathways required to identify donors, collect samples and donations, and test and manufacture HT CCP units for patients in need. The identified pathway was then converted into a deterministic spreadsheet‐based economic model (in MS Excel) to synthesize healthcare resources and unit costs and estimate the costs per CCP donation, HT CCP donation, and HT CCP unit in the English CCP programme.

A healthcare system perspective was adopted where resources consisted of all materials, facilities, equipment and staff used to collect and process plasma donations. Unit costs refer to the cost of providing a single unit of resource, based on prices paid by the public healthcare providers of the plasma collection programmes, where available. Where relevant and feasible we included indirect costs (e.g., equipment and staff shared across healthcare services) and general overheads (e.g., cost of water, electricity and heating, national insurance and pension contributions for staff costs).

In costing analyses, we identify, measure and value resource and cost components to varying levels of detail. Resources can be identified and measured in great detail (‘micro‐costing’), or at a more aggregated level (‘gross‐costing’). Similarly, expenditure data can be collected at an organization level and apportioned to units of activity (‘top‐down costing’), or resource use can be measured for individual patients, valued using unit costs and summed to generate total costs per patient (‘bottom‐up costing’).^9^

The English economic model was key to informing data collection in other European countries and estimating the cost per HT CCP donation and HT CCP unit in those jurisdictions. In England, we obtained detailed information on pathways, resource use and unit costs (e.g., volume and unit costs of tests, manufacturing, staff levels and their costs by activity performed) allowing a ‘top‐down micro‐costing’ approach.^9^ In the remaining European countries, we obtained aggregate resource use items and costs (e.g., total staff costs, total expenditure on apheresis machines) allowing a ‘top‐down gross‐costing’ approach. We also used the same ‘top‐down gross‐costing’ approach in England to validate the findings from the more detailed approach and allow comparisons with other European countries. After the main CCP programme ended in England some costs were later recouped by repurposing CCP for diagnostics. When comparing costs between countries, this cost recovery was factored into the data presented.

To compare costs between countries, we adjusted costs in Euros for cost‐of‐living differences using purchasing power parities (PPPs) across the 28 states of the EU, using the latest figures at the time of analysis (2019).^10^

### 2.1 Defining a model framework for the collection and manufacture of CCP

A national programme for CCP in England was implemented by NHSBT between April 2020 and March 2021, to support two RCTs, RECOVERY and REMAP‐CAP.^2^, ^3^ We mapped the pathways for collecting and manufacturing CCP over time by gathering information through meetings with staff across NHSBT, and identified the resources required at each stage in the pathway to providing CCP to hospitals in the UK.

Phase 1 of the programme (April–June 2020) used existing infrastructure and staff in 33 collection sites. Six new ‘pop‐up’ CCP donor centre sites were established to increase capacity and infrastructure for Phase 2 (June 2020 to March 2021). The CCP programme stopped on 18 March 2021, when two of the ‘pop‐up’ sites closed while others returned to pre‐pandemic activity or to collection of plasma for immunoglobulins. Our work incorporates changes within and between the two phases of the programme.

Resource use items associated with the CCP collection programme in England were divided into five categories: (1) outreach and administrative duties, (2) sample testing (sample‐first and off‐session), (3) CCP collection, (4) manufacturing, and (5) other. Each category was associated with several activities, for example calls and appointments made in outreach and administrative duties. Volume data were obtained from the CCP national programme between April 2020 and March 2021. Volumes of activity were multiplied by the respective resource use and unit cost to estimate variable monthly costs. These were added to the fixed costs to estimate the total costs of the CCP programme. We then divided the total cost by the total number of confirmed HT‐donations. In the absence of data, we assumed the number of HT units per donation to be the same as the number of units obtained without waste per donor following the collection procedure. For further details on fixed and variable costs and associated activities, see supplementary material Tables S1 and S2.

Costs were separated into ‘personnel costs’ and ‘other costs’. We used data on staffing requirements for the new centres established in Phase 2 to cost staff time for ‘sample‐first’ testing (in donor centres) and CCP collection. Salaries were estimated using the midpoint of the NHS Agenda for Change (AfC) bands.^11^ The staffing model for the new centres were: 1 Donor Centre Manager (AfC band 7), 4 nurses (AfC band 6), and either 18 donor carers (AfC band 3) for 6 bed sites, or 25 donor carers for 9 bed sites. ‘Other costs’ included equipment purchased, storage facilities, marketing campaigns, tests and consumables.

The purchase costs of donation chairs were converted into equivalent annual costs, assuming a lifetime of 7.5 years and using a discount rate of 3.5%. Overheads at 20% were added to sampling (in donor centres), CCP collection and manufacturing; overheads were assumed to be included in marketing and home sampling costs provided.

### 2.2 Data collection from blood establishments across Europe

We identified blood establishments within European countries that had set up CCP programmes. Based on the UK test case, we developed a questionnaire covering the CCP collection pathways and associated costs, and the demand and supply of CCP. Information was collected from the start of the CCP programme in 2020 to the end of 2021 on:

- How the CCP collection sites were established—the need, or not, for new infrastructure, and changes during the pandemic.
- The process of defining a HT unit—timelines of screening strategies, definitions of HT, types of assays used.
- The route to donor collection—samples tested first before recalling donor to collect units, or recruitment to donate and test all when collected.
- Number of COVID‐19 cases and hospitalizations during the pandemic to understand supply/demand for CCP.
- Volume of samples collected and volumes of donations collected during first, second and subsequent waves to better understand outreach and success rates of the programme.
- Costs associated with CCP collection—costs of additional infrastructure, unit costs of equipment and consumables, the total cost of the programme.
- Aftermath of CCP programme—reallocation of resources/investment to other programmes.

The questionnaire was designed by two health economists, with input from NHSBT colleagues, and sent to blood establishments in England, France, Germany, Italy, the Netherlands and Switzerland in March 2022 for initial feedback, before being rolled out to an additional 15 countries (18 blood establishments) across Europe by the European Blood Alliance (in May 2022). These data were used to estimate the costs of CCP, compare collection programmes and assess the variation in unit costs across Europe. Analyses were based on questionnaire responses; no attempt was made to impute missing questionnaire data, since we did not have related variables that could be used to predict missing values.

## 3 RESULTS

### 3.1 Collection and manufacturing CCP pathways in England

At the start of the CCP programme eligible donors were booked in for a donation appointment and samples were taken for antibody‐testing as part of the donation (‘straight to donation’). After Phase 1, antibody‐screening before donation was introduced since CCP often had insufficient anti‐SARS‐CoV‐2 levels for the clinical trials, and the median anti‐SARS‐CoV‐2 concentrations in plasma collected from male donors were twice that from female donors. Hence, from August 2020, only males with a positive polymerase chain reaction (PCR) result for SARS‐CoV‐2 were booked ‘straight to donation’. Untested males and females with a positive PCR result provided samples first for antibody‐testing and returned to donate if the sample demonstrated sufficient antibody levels (‘sample‐first’).

Towards the end of Phase 2, ‘sample‐first’ was phased out into ‘off‐session’ testing offered only to men where samples were taken outside donor centres at the donor's home using commercial kits. Individuals returning HT samples were then invited for donation. This co‐existed with males being called straight to session and clearance of the ‘sample‐first’ backlog (see Figure 1). Figure 2 presents the pathway for in‐session testing and CCP collection during Phase 2.

**FIGURE 1:**
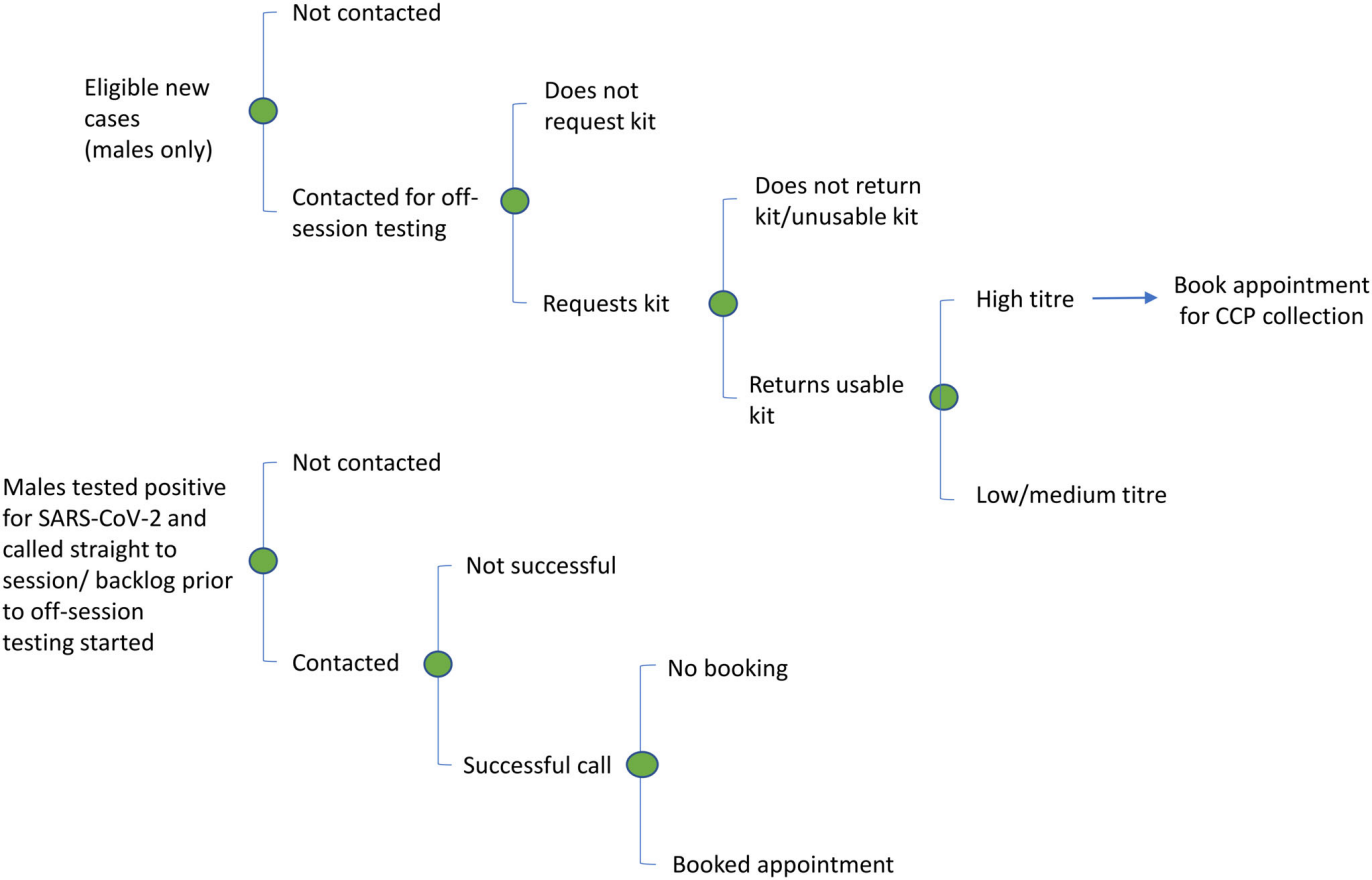
Screening strategy implemented during Phase 2 in a blood establishment in England. CCP, COVID‐19 convalescent plasma.

**FIGURE 2:**
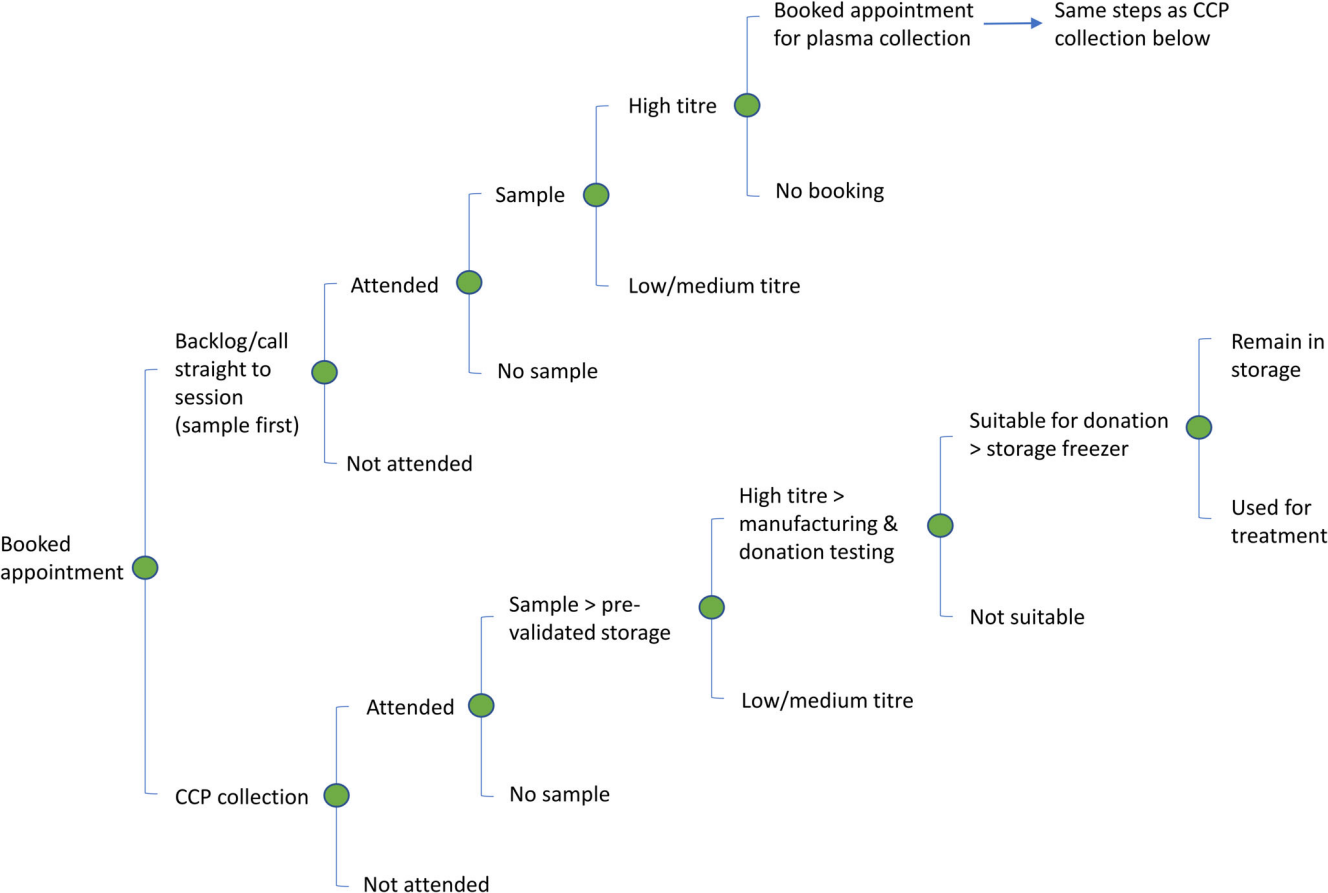
‘Sample first’ (straight to session testing) and CCP collection during Phase 2 in a blood establishment in England. CCP, COVID‐19 convalescent plasma.

### 3.2 Total and component costs of CCP provision in England

The cost per donation, HT donation and HT unit were determined using the budget for the overall CCP programme, for phases 1 and 2 (top half of Table 1, top‐down gross‐costing of CCP provision in England). Costs per donation/unit were significantly lower in Phase 2 and were 60–65% of the costs in Phase 1 of the programme.

**TABLE 1:** Top‐down gross‐costing and micro‐costing of CCP provision in England.

	Cost per donation (Euro PPP)^a^	Cost per HT donation (Euro PPP)^a^	Cost per HT unit (Euro PPP)^a^
Top‐down gross‐costing of CCP provision in England			
Overall (21/04/2020–14/03/2021)	1102	3490	1796
Phase 1 (21/04/2020–12/07/2020)	1550	5129	2700
Phase 2 (13/07/2020–14/03/2021)	1011	3173	1626
Top‐down micro‐costing of each component of CCP provision in England across the whole time of the programme			
Marketing	61	192	99
Sample testing (in or off‐session)	57	179	92
CCP collection	656	2078	1069
Manufacturing/storage	63	199	102
Total	836	2648	1362

Abbreviations: CCP, COVID‐19 convalescent plasma; HT, high‐titre; PPP, purchasing power parities.

^a^ Costs in Euros adjusted for cost‐of‐living differences.

The costs per donation, HT donation and HT unit based on top‐down micro‐costing across the whole time of the programme are 24% lower than those based on the overall budget (bottom half of Table 1 compared to the top half), that is the micro‐costing underestimates costs by 24%. However, the micro‐costing provides estimates of the different components of total cost. From the bottom half of Table 1, the major cost, and key driver of the overall cost of the programme, is the cost of collecting HT CCP. Since only 32% of donations were HT, the cost per HT donation is three times the cost of any CCP donation. Regardless of this, the cost of CCP collection is still the key cost driver.

Table 2 presents the different components of costs for later periods of the programme, which are often lower than costs across the whole time of the programme (bottom half of Table 1). Table 2 also examines the relative costs of when samples were tested; ‘off‐session’ testing was approximately a third the cost of ‘sample‐first’ testing to get a HT sample. The unit cost of ‘sample‐first’ testing for HT CCP was similar to costs for testing after attending the donor centre ‘straight to donation’, or latterly for ‘off‐session’ testing. The costs of ‘sample‐first’ and ‘off‐session’ testing were 55 and 52 Euro PPP per sample respectively. Samples taken at the donor centre were from any eligible donors whilst off‐session sampling only recruited male donors who were more likely to donate HT CCP. Thus, 20% of samples taken at the donor centre were HT, compared to 51% of samples obtained off‐session; the cost per HT sample from off‐session testing was less than half the cost from samples at the donor centre.

**TABLE 2:** Top‐down micro‐costing of each component of CCP provision in England for later periods of the programme.

Component	Average cost over the time period (Euro PPP)^a^		Time period
Marketing	99	per HT unit	20 Apr 2020–14 Mar 2021
Sample testing (in session)	279	per HT sample	2 Nov 2020–14 Mar 2021
Sample testing (off‐session)	101	per HT sample	14 Jan 2021–15 Feb 2021
CCP collection	799	per HT unit	2 Nov 2020–14 Mar 2021
Manufacturing/storage	86	per HT unit	2 Nov 2020–14 Mar 2021

Abbreviations: CCP, COVID‐19 convalescent plasma; HT, high‐titre; PPP, purchasing power parities.

^a^ Costs in Euros adjusted for cost‐of‐living differences.

### 3.3 Plasma collection programmes across Europe

We obtained data from nine European countries concerning their plasma collection programmes (including England). At the start of the pandemic, the infrastructure for CCP collection was established in four countries (France, Italy, Portugal and Sweden), but it took three to 12 weeks to set up the infrastructure in the remaining five countries (Belgium, England, Finland, Norway and Switzerland).

Table 3 describes the key features of the plasma collection programmes across Europe. At the time of the survey, programmes in three countries were ongoing (France, Italy and Switzerland), but had very largely halted in all other countries. The volume of the CCP programme in England was 5‐ to 10‐fold higher than in other countries. The proportion of donations collected that were of HT varied significantly between countries, from 31% (Sweden) to 100% (Belgium). All countries were able to meet the demand for CCP when the number of patients requiring treatment increased during surges of infection except one (and not applicable for another). All countries used CCP in RCTs; some also issued it for compassionate use for severely ill patients.

**TABLE 3:** Convalescent plasma collection programmes across Europe.

	Belgium	England	Finland
Regional/national data	Regional	National	National
Infrastructure for CP collection already in place; if no, time to set up	No (hardware in place, but not procedures); 3 weeks	No; 12 weeks	No; 12 weeks
Time period of CCP programme	16/04/2020–26/05/2021	21/04/2020–18/03/2021, restarted small scale 15/11/2021	30/07/2020–14/01/2021
Time period of data	16/04/2020–26/05/2021	21/04/2020–14/03/2021	30/07/2020–14/01/2021
Number of CCP donations collected (categories in 5000 bands)	0–5000	50,000 – 55,000	0–5000
Percentage of donations collected that were HT	100%	32%	40%
Purpose of CCP collection	Research (clinical trial), monitored emergency use	Research (clinical trial), stockpiling, providing standards or reagents for assays	Research (clinical trial)
Severity of COVID‐19 experienced by the donor specified for CCP collection	Donor had been hospitalized, was hospitalized and required oxygen, required mechanical ventilation or was in an intensive care unit	No severity specified	Initial criteria, donor hospitalized, but relaxed when recovered/vaccinated donors included
Sample tested for antibodies against SARS‐CoV‐2 before collecting CCP units?	Yes	Yes	No
If yes, proportion of samples that led to CCP donation	28%	15%	Not applicable
Threshold for HT CCP based on local non‐standardized testing	≥1:160 titre by neutralization assay (50% cut‐off)	Euroimmun >6 by immunoassay, (considered equivalent to neutralization titre of >1:100 wildtype variant)	80% by neutralization assay

	France	Italy	Norway
Regional/national data	National	Regional	Regional
Infrastructure for CP collection already in place; if no, time to set up	Yes	Yes	No; 6 weeks
Time period of CCP programme	07/04/2020 – ongoing (collection interrupted Jun‐Oct 20, and 2 weeks in early Dec 21)	17/03/2020–ongoing	15/05/2020–15/02/2022
Time period of data	07/04/2020–end 2021	17/03/2020–end 2021	15/05/2020–end 2021
Number of CCP donations collected (categories in 5000 bands)	5000‐10,000	0–5000	0–5000
Percentage of donations collected that were HT	65%	60%	40% of units collected HT
Purpose of CCP collection	Research (clinical trial), monitored access treatment, stockpiling	Research (clinical trial), treatment, stockpiling	Research (clinical trial), treatment
Severity of COVID‐19 experienced by the donor specified for CCP collection	No severity specified	No severity specified	No severity specified
Sample tested for antibodies against SARS‐CoV‐2 before collecting CCP units?	No	Yes	Yes
If yes, proportion of samples that led to CCP donation	Not applicable	50%	Not known
Threshold for HT CCP based on local non‐standardized testing	>40 titre by neutralization assay (99% cut‐off) to Oct 2020, titre>80 thereafter. Immunoassay for all CCP (Euroimmun, ratio >6 to 8 neutralization initially, then only for validation studies)	>1:160 by neutralization assay	60% ACE2‐inhibition

	Portugal	Sweden	Switzerland
Regional/national data	National	Regional	Regional
Infrastructure for CP collection already in place; if no, time to set up	Yes	Yes	No (plasmapheresis in place, but not procedures for CP); 4 weeks
Time period of CCP programme	04/04/2021–04/05/2021 (although samples taken in 2020)	07/04/2020–24/06/2021	22/05/2020–ongoing
Time period of data	2020–04/05/2021	07/04/2020–24/06/2021	22/05/2020–06/2022
Number of CCP donations collected (categories in 5000 bands)	0–5000	0–5000	0–5000
Percentage of donations collected that were HT	Not reported	31%	84%
Purpose of CCP collection	Research (clinical trial), treatment, stockpiling	Research (clinical trial), treatment, stockpiling	Research (clinical trial), treatment
Severity of COVID‐19 experienced by the donor specified for CCP collection	Donor was not hospitalized	No severity specified, donor was not hospitalized	Donor was not hospitalized
Sample tested for antibodies against SARS‐CoV‐2 before collecting CCP units?	Yes	Yes	Yes
If yes, proportion of samples that led to CCP donation	26%	Not reported	05/2020–03/2021: 30%. Since 06/2021: >95%
Threshold for HT CCP based on local non‐standardized testing	In 2020, neutralizing antibodies titre ≥1:80. In 2021, neutralizing antibodies titre ≥1:160.	04–11/2020: Euroimmun>2,0. 12/2020–01/2021: Euroimmun>4,0. 01/2021: Euroimmun>40 (same level as 12/2020, new version displayed resultx10 compared to previous version). 02/2021 onwards: Phadia >(50‐)60 for blood groups O/A, >(30‐)40 for B/AB. [Phadia 60~= Euroimmun 120.]	Titre >4 S/CO by Elisa/EuroImmun (05/2020–03/2021); Titre >8 S/CO by ELISA/EuroImmun (from 06/2021)

Abbreviations: CCP, COVID‐19 convalescent plasma; CP, convalescent plasma; HT, high‐titre.

Seven out of nine countries either did not specify a severity level of COVID‐19 experienced by the donor as a selection criterion for CCP collection or specified that the patient was not hospitalized. One country (Finland) dropped their initial criteria that required donors to be hospitalized, while another (Belgium) required donors to have been hospitalized. Seven out of nine countries tested samples to measure antibodies against SARS‐CoV‐2 before collecting CCP units. The proportion of donors tested for anti‐SARS‐CoV‐2 titres who completed CCP donation varied considerably between 15% (England) and 95% (Switzerland).

### 3.4 Costs in other European countries

Total cost data were obtained from four countries. Table 4 reports total costs per HT donation and per HT unit for available countries across Europe, and the elements included within costs. Estimates are very different: the cost per HT CCP unit varies from €198 to €1749, but what is included within costs also differs significantly. Costs are significantly lower in countries where the infrastructure for CCP collection was in place at the start of the pandemic.

**TABLE 4:** Unit costs and elements included based on total expenditure in a sample of CCP programmes in Europe.

Country	*A*	*B* ^a^	*D*	*F*
Cost (Euro PPP)^b^ per HT donation	265	‐	3399	2353
Cost (Euro PPP)^b^ per HT unit	265	198	1749	807
Elements included in total costs of the CCP programme	Assays for identifying HT, Manufacturing, Transport and storage	Assays for identifying HT, Manufacturing, Pathogen reduction, Hepatitis E NAT testing, Cost savings from repurposing CCP	Marketing campaigns, Investment in infrastructure, Purchase/use of apheresis machines, Assays for identifying HT, Manufacturing, Cost savings from repurposing of CCP	Investment in infrastructure, Purchase/use of apheresis machines, Assays for identifying HT (partially), Manufacturing, Transport and storage
Pre‐existing infrastructure	Yes	Yes	No	No

Abbreviations: CCP, COVID‐19 convalescent plasma; HT, high‐titre; PPP, purchasing power parities.

^a^ Estimate based on cost per HT CCP unit provided, rather than the total costs of the programme and total units produced.

^b^ Costs in Euros adjusted for cost‐of‐living differences.

Table 5 compares unit costs of equipment, consumables and manufacturing across Europe using gross‐costing methods. Annual costs for apheresis machines varied significantly between €391 and €7892, suggesting very different contracts; there was less variation for donation chairs (€512–€771). Costs of apheresis collection kits were €25–€180, assays €4–€70 and manufacturing per unit €90–€185.

**TABLE 5:** Comparison of costs of equipment, consumables and manufacturing across Europe.

Country	Infrastructure in place at start of pandemic	Annual equivalent costs of equipment (Euro PPP)^a^		Unit costs of consumables and manufacturing (Euro PPP)^a^		
		Apheresis machine	Donation chair	Apheresis collection kit	All assays	Manufacturing per unit
A	Yes	5009	771	180	70	95
B	Yes	391			5	185
C	Yes				4	
D	No	7892	512	25	27	90
E	No	2611				
F	No			32		161
G	No				44	

Abbreviation: PPP, purchasing power parities.

^a^ Costs in Euros adjusted for cost‐of‐living differences.

## 4 DISCUSSION

This is the first estimate of comparative costs of CCP collection across several countries. The micro‐costing of CCP production in England suggests that the key driver of costs was activities required for the collection of HT CCP; an area that needs to be optimized. We focused on the production of HT units since this was the desired product, although we incorporated costs recovered from non‐HT units in our comparisons across Europe. The scale of CCP programmes across Europe and their costs varied significantly. Costs were lower in countries which built on ongoing plasma collection programmes and where the infrastructure for CCP collection existed at the start of the pandemic, compared to countries starting a programme from scratch. As blood establishments across Europe implement or increase plasma collection, we would expect these services to be more prepared for collecting specific CP in a future pandemic.

Average costs of HT CP will probably change during the implementation and management of a CP programme, as countries improve their programme's efficiency. In England, the cost of an HT CCP unit in Phase 2 was 60% of the cost in Phase 1. Our detailed top‐down micro‐costing approach enabled us to better understand the key drivers of costs. Collecting large volumes of CCP first and then testing for high levels of neutralizing antibodies was more expensive than selecting donors with HT antibodies (tested by a small blood sample) prior to scheduling collection. Later even more efficient ways of testing (home kits) were introduced. Most components of costs were lower in later periods of the programme, when new and more efficient pathways and procedures were applied and volumes of collection were greater. The volume of CCP collection was a key cost driver as the programme evolved; however, it was not possible to separate the cost of establishing a CCP programme from the cost of CCP production from the data collected.

The quality of HT CP produced will impact its effectiveness and costs. Methods used to define HT CCP varied during the COVID‐19 pandemic with re‐evaluation of the threshold used. Detailed analyses within the SUPPORT‐E consortium (https://www.support-e.eu/) have shown that there is not a linear relationship between standardized neutralizing antibody (nAb) potency (standardization against international units) and neutralization titre across different countries and laboratories.^12^ For example, one country using a neutralizing titre of 1:100 had a nAb potency of 108 IU/mL, yet another region with a neutralizing titre of 1:80 had a nAb potency of 409 IU/mL. There is significant variability in the assays used to assess SARS‐CoV‐2 neutralization and/or antibody content, and estimates of antibody potency in CCP. Moreover, the proportion of donors selected for HT plasma production may vary during the course of a pandemic as new variants emerge. Therefore, it was not possible to normalize the estimated costs of HT CCP for SARS‐CoV‐2 potency (that is the neutralizing antibody titre against the circulating variant) to standardize that aspect of the HT CCP unit costs, to make costs from different countries more comparable.

Another aspect of the quality of HT CP is pathogen reduction, which reduces bacterial and viral contamination of components and is an additional CP‐manufacturing cost. Pathogen reduction limits the ability to repurpose CP, directing these units exclusively for clinical use, as pathogen‐reduced units cannot be sold for fractionation or diagnostics. Countries without pathogen reduction have the option to repurpose CP and recoup some of the costs of manufacturing CCP. Indeed, NHSBT (England) recouped some of the total costs of its CCP programme by selling plasma (which is factored into the costs presented), so this could be considered for future pandemics. Blood establishments in six of the nine countries responding to the survey used pathogen reduction, so could not repurpose CCP for fractionation. However, repurposing CP for general clinical use, if the neutralizing antibody level was below the threshold or the unit was nearing the end of its shelf‐life, was widely used. If England had been able to sell CCP units before expiry as standard plasma for direct clinical use, it would have generated 1.5 times more income than was recouped from repurposing the plasma for diagnostics.

Outcomes from the RECOVERY and REMAP‐CAP trials suggested that CCP would be most beneficial for participants unable to generate antibodies against SARS‐CoV‐2.^13^, ^14^ If this were true for other viral infections then less CP would be needed to treat a specific population. At the time of writing the role of CCP for treating sub‐groups of patients with COVID‐19 had not been established, hence the focus here on estimating the costs of setting up and running CCP programmes across Europe, rather than a budget impact analysis, incorporating patient outcomes.

In any future pandemic, start‐up costs may include setting up a plasma apheresis programme, irrespective of the income status of a given country. Equity concerns could be addressed by ‘sharing’ resources (here CCP) across EU countries, which is one of the objectives of the SoHO Regulation (Regulation on Standards of Quality and Safety for Substances of Human Origin (SoHO) Intended for Human Application).^15^ To improve their preparedness, countries could develop plasma apheresis programmes if not yet in place, and develop programmes that allow for CCP (or equivalent) to be used for general transfusion purposes if not used for the primary purpose. Analyses as described here will provide financial indicators to inform pandemic preparedness strategies that continue to be developed in Europe and in the UK.^16^, ^17^ The EU could use the information presented to target financial support (both for preparedness and at the time of crisis), and to inform the development of preparedness plans by the SoHO Coordination Board (SCB).

A challenge with these analyses was the availability of data. Collecting detailed data in England was a challenge once the initial and larger programme had closed and staff had moved on to different roles. For donor centre activities, we could not separate the staff time associated with in‐session sampling (‘sample first’) from CCP collection, so costs of staffing both activities were included within CCP collection costs; thus costs of ‘in‐session’ samples were underestimated, whilst costs of CCP collection were overestimated. All centres were costed as though they were new centres, rather than a mix of new and existing centres. Our micro‐costing approach underestimated total costs by 24%, compared to the actual budget for the programme. However, the distribution of costs across activities obtained via micro‐costing provided key insights to its major drivers. There were challenges with the top‐down gross‐costing data collection too. Many services across Europe completing the questionnaire reported difficulties obtaining all the information requested, hence some missing data on returned questionnaires, and the response rate of 43% (9/21 countries). In any future pandemic, efforts should be made to record and retrieve such data to facilitate a greater depth of economic analysis of plasma collection. A further limitation is that it was not possible to report variability or confidence intervals around cost estimates.

We have shown, with the data available, that costs are lower in countries where the infrastructure for CCP collection was in place at the start of the pandemic. A sample‐first policy is less costly than collecting plasma and then testing to identify HT units, so this is recommended for future CP programmes. Expenditure surrounding CCP collection is the key cost driver, so any additional steps to increase the proportion of donations that are HT would be highly beneficial and improve efficiency. Repurposing of CP can be used to recoup costs, therefore alternative uses for plasma that is not HT should be sought—for example for clinical use, fractionation or diagnostics if possible. In future, timely efforts to capture prospectively granular data on the resources required, costs of the collection and use of CP by blood establishments will be essential to improve budget planning and preparedness to treat infected patients during a global pandemic.

## FUNDING INFORMATION

This work was conducted as part of SUPPORT‐E (SUPporting high quality evaluation of COVID‐19 convalescent Plasma thrOughoutT Europe), which was supported by funding from the European Union's Horizon 2020 research and innovation programme under grant agreement No. 101015756.

## CONFLICT OF INTEREST STATEMENT

The authors have disclosed no conflicts of interest.

## Supporting information


**Table S1.** Resource use items and costs of the CCP collection programme in a blood establishment in England.
**Table S2**. CCP programme activities for which throughput data were sought from a blood establishment in England.

## Data Availability

Data is not available for sharing, as we do not have permission to share beyond the study team.
