# Supplementary material for: Assessing the financial resources required to establish convalescent plasma collection programmes across Europe in response to a pandemic
Source: Transfusion. 2026 Oct 1;66(11):2155–66. doi: 10.1111/trf.70323 (PMC13753061; doi:10.1111/trf.70323)
Supplement: Supplementary file 1 [file TRF-66-2155-s001.docx]

**Supplementary Material**

Costs were defined as either “fixed” or “variable” costs. Fixed costs were those which did not change even if more donors were seen and more samples collected and processed. Variable costs were those which changed with the volume of donors and samples processed.

Table S1: Resource use items and costs of the CCP collection programme in a blood establishment in England

|  | **Category** | **Variable costs** | **Fixed costs** |
| --- | --- | --- | --- |
| (1) | Outreach and administrative duties | Inbound and outbound calls from/to (potential) donors, SMS and blood/organ training | Marketing campaign, IT resource/project management, website hosting |
| (2a) | Sample testing: sample-first, straight to session | Phlebotomy consumables, tests (Euroimmun and antibody assay) | Donation chairs, staff, overheads |
| (2b) | Sample testing: off-session testing (men only) | Sample sent and returned, sample sent and not returned | None |
| (3) | CCP collection | Phlebotomy consumables, tests (Euroimmun and antibody assay), reagent and harness by procedure | Donation chairs, apheresis machines, staff, overheads |
| (4) | Manufacturing | Consumables | Staff, storage facilities, overheads |
| (5) | Other | Transport of CCP from storage to use | Building leases |

CCP, COVID-19 convalescent plasma

Table S2: CCP programme activities for which throughput data were sought from a blood establishment in England

|  | **Category** | **Activity** |
| --- | --- | --- |
| (1) | Outreach and administrative duties | Calls made  Successful calls made  Appointments made (outbound/inbound)  Sampling and collection dates booked |
| (2a) | Sample testing: sample-first, straight to session | Samples booked  Sampling attendances  Successful samples  HT samples |
| (2b) | Sample testing: off-session testing (men) | Kits requested  Kits returned  HT samples |
| (3) | CCP collection | Donations booked  Donations collected  Total procedures  Total units collected  Donations confirmed as HT |
| (4) | Manufacturing | Donations confirmed as HT sent for manufacturing |
| (5) | Other | Transport of CCP from storage to use |

CCP, COVID-19 convalescent plasma; HT, high-titre
